# Accurate Biomolecular Simulations Account for Electronic Polarization

**DOI:** 10.3389/fmolb.2019.00143

**Published:** 2019-12-04

**Authors:** Josef Melcr, Jean-Philip Piquemal

**Affiliations:** ^1^Groningen Biomolecular Sciences and Biotechnology Institute and the Zernike Institute for Advanced Materials, University of Groningen, Groningen, Netherlands; ^2^Laboratoire de Chimie Théorique, Sorbonne Université, UMR7616 CNRS, Paris, France; ^3^Institut Universitaire de France, Paris, France; ^4^Department of Biomedical Engineering, The University of Texas at Austin, Austin, TX, United States

**Keywords:** molecular dynamics simulations, electronic polarization, electronic continuum correction, biomolecules, phospholipids, amino acids, nucleic acids, ions

## Abstract

In this perspective, we discuss where and how accounting for electronic many-body polarization affects the accuracy of classical molecular dynamics simulations of biomolecules. While the effects of electronic polarization are highly pronounced for molecules with an opposite total charge, they are also non-negligible for interactions with overall neutral molecules. For instance, neglecting these effects in important biomolecules like amino acids and phospholipids affects the structure of proteins and membranes having a large impact on interpreting experimental data as well as building coarse grained models. With the combined advances in theory, algorithms and computational power it is currently realistic to perform simulations with explicit polarizable dipoles on systems with relevant sizes and complexity. Alternatively, the effects of electronic polarization can also be included at zero additional computational cost compared to standard fixed-charge force fields using the electronic continuum correction, as was recently demonstrated for several classes of biomolecules.

In molecular dynamics simulations, the interactions between molecules are described with approximate potentials known as force fields that mimic the true Born-Oppenheimer energy hypersurface. Among these methods, pairwise additive potentials are very popular for modeling biomolecules such as proteins, lipids or nucleic acids (Ponder and Case, 2003; Lopes et al., 2015). The current standard force fields (Huang and MacKerell, 2013; Maier et al., 2015; Robertson et al., 2015), however, neglect important physical many-body effects such as the electronic polarization, charge transfer, or many-body dispersion (cited in decaying magnitude order) (Kleshchonok and Tkatchenko, 2018). Although such models have provided valuable insight into many phenomena from various fields including biology, chemistry, biophysics, or material sciences, there are several important cases in which accounting for polarizability is crucial.

## Pitfalls of Non-polarizable Force Fields

The limited predictive accuracy of non-polarizable force fields led the molecular modeling community to develop new generation “polarizable” force fields (Gresh et al., 2007; Jorgensen, 2007; Stone, 2013; Shi et al., 2015; Piquemal and Jordan, 2017; Kleshchonok and Tkatchenko, 2018; Martinek et al., 2018; Melcr et al., 2018, 2019; Antila et al., 2019; Jing et al., 2019) able to include the missing physics with a special focus on the polarizability effects. Although such techniques are now widely used in fields studying highly charged ionic liquids (Bedrov et al., 2019), their application cannot be limited only to such extreme cases. For instance, neglecting the effects of the electronic polarizability in important biomolecules like amino acids, nucleic acids, and phospholipids affects the structure of proteins (Jiao et al., 2008; Shi et al., 2009; Duboué-Dijon et al., 2018a), DNA (Babin et al., 2006), and membranes (Harder et al., 2009; Catte et al., 2016; Melcr et al., 2018) having a large impact on interpreting experimental data (Hauser et al., 1977; Eisenberg et al., 1979; Kurland et al., 1979; Feigenson, 1986; Mattai et al., 1989; Roux and Bloom, 1990, 1991; Böckmann and Grubmüller, 2004; Lund et al., 2008; Vacha et al., 2009; Berkowitz et al., 2012; Melcrová et al., 2016; Javanainen et al., 2017; Magarkar et al., 2017) as well as building coarse grained models. Importantly, these results show that the electronic polarization yields non-negligible effects also at overall neutral molecules (Gresh et al., 2007; Melcr et al., 2018).

Secondary structure of proteins is to a large extent determined by an intricate network of hydrogen bonds. The description of hydrogen bonds in standard force fields, however, does not contain important contributions, e.g., from polarization and partially covalent character (Babin et al., 2006). It was demonstrated in many cases including structure of water (Dang, 1998), binding of ligands to proteins (Friesner, 2005; Jiao et al., 2008), and protein folding and unfolding (Morozov et al., 2006; Freddolino et al., 2010; Piana et al., 2011, 2014; Huang and MacKerell, 2014; Lemkul et al., 2016; Célerse et al., 2019) that polarizability contributes significantly to the accuracy of simulations of structures with hydrogen bonds. Also, salt bridging between amino acids is likely overestimated in strength when the effects of polarization are not included (Friesner, 2005; Vazdar et al., 2013; Debiec et al., 2014; Ahmed et al., 2018; Célerse et al., 2019; Mason et al., 2019). For instance, the interaction of acidic side chains of glutamate and aspartate with cations is overestimated in strength in classical non-polarizable force fields (Patel et al., 2009; Duboué-Dijon et al., 2018a), while treatment of polarizability in solvent relaxation affects salt bridge dissociation (Célerse et al., 2019). Taken together, the secondary and tertiary structural arrangements in the simulations of proteins are likely biased to certain preferred configurations due to the lack of polarizability depending on the chosen parametrization strategy (Freddolino et al., 2009, 2010; Piana et al., 2011, 2014).

Membrane proteins form a large part of cellular proteome and are in direct contact with amphiphilic cellular membranes, which influence their structure and activity (Lee, 2004). Membranes themselves are crucial cell organelles which define the inner resp. outer cellular environment. They are predominantly composed of amphiphilic phospholipids, which self-assemble into stable bilayer structures (Harayama and Riezman, 2018). The force fields for phospholipids have been tuned to the level that the simulations of commonly used simplified model lipid membranes can reproduce a large variety of experimentally measured properties, phenomena and structural features including lipid self-diffusion, x-ray scattering patterns, bilayer thickness, area per phospholipid, and acyl chain order parameters (Pluhackova et al., 2016).

This could make an impression that the currently available non-polarizable lipid force fields provide comparable accuracy to the models with explicit polarization at a fraction of the computational cost. While the non-polarizable models yield accurate results in many cases (Lucas et al., 2012; Chowdhary et al., 2013a), simulation studies have revealed that such models gradually lose their predictive accuracy with increasing complexity beyond model systems used during their parametrization, e.g., when membranes are put into contact with buffers of physiological ionic strengths (Catte et al., 2016). For instance, improvements in the electrostatics of phospholipid membranes have a great impact on the membrane dipole potential, permeation of water through membranes, and viscosity of organic liquids (Harder et al., 2009; Venable et al., 2019). Moreover, the interactions between phospholipids and cations, especially divalent cations like calcium, are overestimated in the classical non-polarizable models (Catte et al., 2016; Melcr et al., 2018; Antila et al., 2019).

In general, the structure of divalent cations complexes that are widespread in biosystems is traditionally problematic in non-polarizable simulations (Kohagen et al., 2015). In contrast, simulations with explicit or implicit treatment of polarization yield comparable accuracy to DFT-based ab-initio calculations and neutron scattering experiments, as was demonstrated for biologically relevant divalent cations Ca^2+^ and Mg^2+^ (Piquemal et al., 2006b; Wu et al., 2010; Martinek et al., 2018). While accounting for the electronic polarization overall improves the predictive accuracy of simulations in general, it is not sufficient in some cases like zinc chloride ion pairing, where more complex physics beyond “mere” electronic polarization is at play (Gresh et al., 2005, 2007; Piquemal et al., 2007; Duboué-Dijon et al., 2018b).

## Implicit Treatment of Electronic Polarization via Electronic Continuum Correction

The necessity of polarizability and screening in modeling lipid bilayers has been an issue from the very beginning of computational modeling of model membranes. The first pioneering works on phospholipid bilayers document the need of including polarizability and extra screening in the development of the first models, which was achieved at that time through an empirical scaling factor for the partial atomic charges of the phospholipids (Egberts et al., 1994). A similar strategy supported by continuum theory was used in the recent developments of phospholipid force fields, which implicitly account for the electronic polarization using Electronic continuum correction (ECC) (Leontyev and Stuchebrukhov, 2009, 2010a; Mason et al., 2012; Pegado et al., 2012; Pluhařová et al., 2013; Martinek et al., 2018). Despite the approximate treatment of the polarizability using ECC, such lipid force fields provide accurate interactions between phospholipid bilayers and cations in agreement with experiments (Melcr et al., 2018). In particular, in the case of the neutral phosphatidylcholine (PC), ECC improved the cation binding affinity for monovalent, and divalent cations reaching agreement with experiments (Melcr et al., 2018), while for negatively charged phosphatidylserine (PS) it has also improved the overall structure of the phospholipid and the interactions with other lipids (Antila et al., 2019; Melcr et al., 2019).

Electronic continuum correction is a very efficient alternative to otherwise computationally demanding explicit modeling of electronic polarization (Bedrov et al., 2019). The accuracy of the ECC method was shown to yield promising results on several polar organic solvents (Leontyev and Stuchebrukhov, 2010b, 2012; Lee and Park, 2011; Vazdar et al., 2013), while it proved to be necessary yet sufficient for an accurate description of the structure of several monovalent and divalent ions in aqueous solutions (Mason et al., 2012; Pegado et al., 2012; Pluhařová et al., 2013). To date, the array of force fields utilizing ECC has grown from a wide range of biologically relevant ions (Kohagen et al., 2014, 2015; Martinek et al., 2018), to protein moieties (Vazdar et al., 2013; Duboué-Dijon et al., 2018a; Mason et al., 2019), and whole phospholipid molecules (Melcr et al., 2018) making realistic simulations of e.g., membrane proteins at physiological ionic conditions possible.

In ECC all particles are assumed to have equal polarizabilities and the electric field and electron density within each particle is homogenous (Leontyev and Stuchebrukhov, 2009). Such approximations simplify the calculations of the polarization to such an extent that it can be simply included in the interactions as a pre-determined charge-scaling factor (Leontyev and Stuchebrukhov, 2009), which is derived from the high-frequency dielectric constant of electrons, ε_*el*_, as $1/\sqrt{\varepsilon_{el}}\approx 0.75$ for aqueous solutions. Importantly, ε_*el*_ is close to 2 for a wide variety of biologically relevant environments meaning that even interfaces like biological membranes do not give rise to large gradients. Despite the coarseness of the approximations, the effects of electronic polarization are described sufficiently well for a variety of biologically relevant molecules in a condensed phase (Duboué-Dijon et al., 2018a,b; Martinek et al., 2018; Melcr et al., 2018). Moreover, ECC accounts for the effects of electronic polarization at zero additional computational cost compared to standard fixed-charge force fields. Although, a new generation of simulation codes performing large scale simulations with explicit polarization models starts to emerge (Lagardère et al., 2018), ECC yields the benefit of employing the widely adopted and already highly optimized codes for classical MD.

The common implementation of ECC via charge rescaling profoundly resembles an empirical scaling factor, which, obviously, reduces the interaction of charged molecules. From both the derived ECC theory (Leontyev and Stuchebrukhov, 2010a) and its applications, which compare ECC to also other methods (Pegado et al., 2012; Martinek et al., 2018), it is however clear that the improvements pertinent to ECC can be attributed to the electronic polarization. For instance, interactions of sulfate anions were directly compared between simulations with ECC, solvent shell model (Rick and Stuart, 2003) and ab-initio calculations (Pegado et al., 2012). This comparison has revealed that ECC performed comparably well to the other methods at a fraction of the computational cost. Moreover, ECC was concluded as preferable over the explicit solvent shell model for sulfate anions as it was closer to the structures from ab-initio calculations (Pegado et al., 2012).

## Capturing Effects Beyond Electronic Polarization

The accuracy of the implicit methods including ECC is limited and gradually becomes inadequate in cases, which do not adhere to the assumed approximations. For instance, the complex electronic structure of Zn^2+^ makes it difficult to capture the ion pairing of zinc chloride with ECC unless specific ad hoc interaction terms between the ions are introduced (Duboué-Dijon et al., 2018b). Hence, resorting to more accurate modeling strategies including explicit polarizable dipoles—or even effects beyond electronic polarization—becomes necessary in such cases.

The AMOEBA force field with explicit polarizable dipoles correctly reproduces water structure around Zn^2+^ in bulk solution and its free energy of hydration, however, it still does not capture the fine details of zinc chloride ion pairing. The reason for that is that Zn^2+^ exhibits considerably large charge transfer effects prefiguring what is happening with transition metals where back-donation effects become important (Gresh et al., 2005, 2007; Piquemal et al., 2007). Simulations then need to utilize more complex polarizable force fields able to separately evaluate the different physical contributions. Indeed, short-range electrostatics in such systems is anything but classical as it is strongly affected by quantum penetration effects in the overlap region (Piquemal et al., 2003, 2006a; Gresh et al., 2005, 2007; Wang et al., 2015). On the contrary, many-body polarization interactions which are usually cooperative (i.e., the total energy being larger that the purely additive contributions) do not behave in such a way (Gresh et al., 2007, 2016; Zhang et al., 2012; Jing et al., 2018). Divalent metal cations in particular locally reverse the physical trends and exhibit net anticooperativity as the total energy becomes smaller than the sum of individual contributions. For example SIBFA (Sum of Interactions Between Fragments Ab initio computed) incorporates a many-body explicit charge transfer (Gresh et al., 2005, 2007; Piquemal et al., 2007) and a penetration correction for electrostatics (Piquemal et al., 2003; Narth et al., 2016), and is able to deal with such difficult systems.

Such effects also exist with variable magnitude in biomolecular simulations, and resorting to more accurate methods employing physics even beyond explicit polarization will be likely required for predictive accuracy in many cases, e.g., metalloproteins, which shall be interesting playgrounds for such modeling (Gresh et al., 2007, 2016; Zhang et al., 2012; Jing et al., 2018). Improvements in capturing correct physics is a general trend in current developments, and besides SIBFA, the AMOEBA force field is gradually evolving into the AMOEBA+ potential, which additionally includes such physical effects (Liu et al., 2019). Moreover, several other general polarizable potentials are emerging (Huang et al., 2017; Das et al., 2019; Rackers and Ponder, 2019) indicating the start of next-generation polarizable force fields development (Piquemal et al., 2006a; Duke et al., 2014; Piquemal and Cisneros, 2016).

## Are polarizable Simulations Computationally Tractable?

This being said the question remains: is there any practically achievable perspective application of such advanced models to meaningfully large simulations of biologically relevant systems?—Certainly yes. If the use of polarizable models has been doomed by their computational cost for years, things have dramatically improved. In terms of computational requirements, the approaches utilizing Drude particles (Lopes et al., 2013) traditionally appeared more feasible compared to explicit point dipole approaches (Lipparini et al., 2014; Lagardère et al., 2015), as their computational cost in standard high-performance codes was higher by a factor 2–4 depending on implementation and reference settings compared to non-polarizable force fields (Jiang et al., 2011), while the explicit point dipoles models were roughly twice slower. However, such models cannot utilize long time-steps because of their use of extended Lagrangian, which practically imposes a speed limit (Wang and Skeel, 2005; Albaugh and Head-Gordon, 2017). In contrast, utilizing advanced algorithms for solving polarization and dynamical integration is possible within explicit point dipole approaches leading to strong speed increases to the performance level of Drude approach (even for higher-level multipolar electrostatics approaches such as AMOEBA) when compared to usual non-polarizable models simulation (Lagardère et al., 2019). However, the numerous available point dipole force fields (AMOEBA, SIBFA etc…) had in practice another handicap besides their computational cost: they were not available in high performance/production codes such as GROMACS or NAMD (Phillips et al., 2002; Van Der Spoel et al., 2005).

This situation has gradually changed in recent years. First, in link with the improved multi-timestep integration, the key mathematical problem of solving the point dipole equations using iterative methods was alleviated using new non-iterative approaches such as the Truncated Conjugate Gradient (TCG-1) (Aviat et al., 2017a,b) that allows for a fix cost evaluation of polarization. When coupled to an analytical evaluation of gradient such an approach fully preserves energy and, hence, allows for long time step simulations. Second, the availability of massively parallel MPI codes able to efficiently use supercomputers using 3D domain decomposition techniques such as Tinker-HP (Lagardère et al., 2018; Jolly et al., 2019) [the high performance engine of the Tinker molecular package Rackers et al., 2018] shed first rays of light at the end of the tunnel leading toward simulations of biologically relevant large systems on long enough timescales with explicit polarization. Moreover, GPU accelerated implementations of AMOEBA in OpenMM (Huang et al., 2018) and Tinker-OpenMM (Harger et al., 2017) are available whereas the support of hybrid (multi)CPUs-GPUs is coming in Tinker-HP (O. Adjoua et al., personal communication).

Overall, methodology has made a key progress and will continue in this direction for all types of polarizable force fields as the accessible computer power quickly increases reducing therefore the computational gap with additive potentials. Whereas specialized highly accurate water potentials based on many-body expansions emerge such as MBPOL (Riera et al., 2019) and allow for a better understanding of fine physical effects in clusters and bulk water, the availability of general polarizable force fields such as AMOEBA offering water (Ren and Ponder, 2003), ions, organochlorine compounds (Mu et al., 2014), proteins and nucleic acids (Shi et al., 2013; Zhang et al., 2018) now enables performing enough sampling to achieve highly accurate and biologically meaningful simulations. The Drude approaches parametrization is expanding as well (Lamoureux et al., 2003; Chowdhary et al., 2013a,b; Lopes et al., 2013). Moreover, accelerated sampling methods start to be applied also to polarizable approaches (Célerse et al., 2019) offering improved simulation capabilities and access to accurate and fast evaluation of free energies of binding thanks to GPUs (Harger et al., 2017). Such capabilities allow to tackle hard systems as in the case of the Phosphate binding mode of the Phosphate-binding protein where it was possible to highlight the critical effect of the buffer solution ending a long standing controversy thanks to free energy computations (Qi et al., 2018).

## Summary: Biomolecular Simulations of the Future are Polarizable

In summary, we have presented several important classes and case studies of biomolecules, where including polarizability is an important factor for the simulation accuracy. Cytosolic environment in cells is mostly composed of water solutions of ions, for which polarizability is necessary for the accurate description of the solvated structure of ions, their pairing and interaction with other biomolecules (Piquemal et al., 2006b; Wu et al., 2010; Mason et al., 2012; Pegado et al., 2012; Pluhařová et al., 2013; Duboué-Dijon et al., 2018a,b; Martinek et al., 2018; Melcr et al., 2018). Polarizability is an important factor for accurate interactions between amino acids, namely salt bridges between them, which are overestimated in strength in current non-polarizable force fields (Friesner, 2005; Vazdar et al., 2013; Ahmed et al., 2018; Célerse et al., 2019; Mason et al., 2019). Moreover, polarizable force fields yield a better description of the hydrophobic effect and hydrogen bond networks in proteins, which to a large extent determine the dynamic structure and conformational changes of proteins (Dill et al., 1995; García-Moreno et al., 1997; Fitch et al., 2002; Morozov et al., 2006; Freddolino et al., 2010; Piana et al., 2011, 2014; Huang and MacKerell, 2014; Lemkul et al., 2016; Célerse et al., 2019; Venable et al., 2019). Polarizability is necessary for accurate structure and interactions of both neutral and charged phospholipids, which constitute a dominant part of cellular membranes (Harder et al., 2009; Catte et al., 2016; Melcr et al., 2018).

The representation of electronic polarization in classical MD simulations can vary largely with Drude and induced point dipoles approaches on one side and continuum approximations on the other (Cieplak et al., 2009; Lopes et al., 2009; Leontyev and Stuchebrukhov, 2011; Schröder, 2012; Baker, 2015; Shi et al., 2015; Lemkul et al., 2016; Bedrov et al., 2019; Jing et al., 2019). With the advances in both computational power together with theory and algorithms it is practically achievable to perform simulations with explicit polarizable dipoles on systems with relevant sizes and complexity (Qi et al., 2018; Bedrov et al., 2019; Lagardère et al., 2019; Loco et al., 2019). In particular, it is currently realistic to perform simulations with explicit polarization at time scales, which are competitive to the standard fixed-charge simulations (Lemkul et al., 2016; Lagardère et al., 2018; Célerse et al., 2019). Moreover, advanced polarizable potentials (e.g., SIBFA, AMOEBA+) including effects even beyond electronic polarization are being actively developed to tackle systems with complex structure like metalloproteins, kinases or ribozymes (Gresh et al., 2007, 2016; Zhang et al., 2012; Jing et al., 2018, 2019; Das et al., 2019; Liu et al., 2019; Rackers and Ponder, 2019). Also, approximate implicit solutions like ECC, which circumvent the computational costs of explicit polarization, gradually gain on popularity and provide a promising solution for a variety of applications in biomolecular simulations (Duboué-Dijon et al., 2018a,b; Martinek et al., 2018; Melcr et al., 2018, 2019; Mason et al., 2019). Finally, as fully variational polarizable embeddings are now possible in hybrid QM/MM molecular simulations (Loco et al., 2016, 2017, 2019), one can expect that hybrid explicit polarization/ECC simulations will be possible in the near future offering a multi-level global treatment of polarization across very large complex molecular systems.

Biomolecules in the real world cannot turn off their polarizability. Hence, molecular dynamics simulations, which aim to give a realistic, robust, and predictive results, cannot afford to neglect this important contribution to the electrostatic interaction. Currently, polarizable force fields for a large variety of biomolecules and simulation codes implementing polarizability exist and are readily available to solve various biophysical problems (Wu et al., 2010; Chowdhary et al., 2013a; Lemkul et al., 2016; Duboué-Dijon et al., 2018a,b; Lagardère et al., 2018; Martinek et al., 2018; Melcr et al., 2018; Zhang et al., 2018; Bedrov et al., 2019; Célerse et al., 2019; Jing et al., 2019; Liu et al., 2019). We expect that the popularity of such approaches will grow and will become a common tool in biomolecular research in the near future.

## Data Availability Statement

All datasets generated for this study are included in the article/supplementary material.

## Author Contributions

All authors listed have made a substantial, direct and intellectual contribution to the work, and approved it for publication.

### Conflict of Interest

The authors declare that the research was conducted in the absence of any commercial or financial relationships that could be construed as a potential conflict of interest.

## References

[B1] Ahmed M. C. Papaleo E. Lindorff-Larsen K. (2018). How well do force fields capture the strength of salt bridges in proteins? PeerJ 6:e4967. 10.7717/peerj.4967 29910983 PMC6001725

[B2] Albaugh A. Head-Gordon T. (2017). A new method for treating drude polarization in classical molecular simulation. J. Chem. Theory Comput. 13, 5207–5216. 10.1021/acs.jctc.7b00838 28965397

[B3] Antila H. Buslaev P. Favela-Rosales F. Ferreira T. M. Gushchin I. Javanainen M.. (2019). Headgroup Structure and Cation Binding in Phosphatidylserine Lipid Bilayers. J. Phys. Chem. B. 123, 9066–9079. 10.1021/acs.jpcb.9b06091 31574222

[B4] Aviat F. Lagardère L. Piquemal J.-P. (2017a). The truncated conjugate gradient (TCG), a non-iterative/fixed-cost strategy for computing polarization in molecular dynamics: Fast evaluation of analytical forces. J. Chem. Phys. 147:161724. 10.1063/1.4985911 29096518

[B5] Aviat F. Levitt A. Stamm B. Maday Y. Ren P. Ponder J. W.. (2017b). Truncated conjugate gradient: an optimal strategy for the analytical evaluation of the many-body polarization energy and forces in molecular simulations. J. Chem. Theory Comput. 13, 180–190. 10.1021/acs.jctc.6b00981 28068773 PMC5228058

[B6] Babin V. Baucom J. Darden T. A. Sagui C. (2006). Molecular dynamics simulations of DNA with polarizable force fields: convergence of an ideal B-DNA structure to the crystallographic structure. J. Phys. Chem. B 110, 11571–11581. 10.1021/jp061421r 16771434

[B7] Baker C. M. (2015). Polarizable force fields for molecular dynamics simulations of biomolecules. Wiley Interdiscipl. Rev. 5, 241–254. 10.1002/wcms.1215

[B8] Bedrov D. Piquemal J.-P. Borodin O. MacKerell A. D. Roux B. Schröder C. (2019). Molecular dynamics simulations of ionic liquids and electrolytes using polarizable force fields. Chem. Rev. 199, 7940–7995. 10.1021/acs.chemrev.8b00763 PMC6620131 31141351

[B9] Berkowitz M. L. Vácha R. Vacha R. (2012). Aqueous solutions at the interface with phospholipid bilayers. Acc. Chem. Res. 45, 74–82. 10.1021/ar200079x 21770470

[B10] Böckmann R. A. Grubmüller H. (2004). Multistep binding of divalent cations to phospholipid bilayers: a molecular dynamics study. Angew. Chem. Int. Ed. 43, 1021–1024. 10.1002/anie.200352784 14966897

[B11] Catte A. Girych M. Javanainen M. Loison C. Melcr J. Miettinen M. S.. (2016). Molecular electrometer and binding of cations to phospholipid bilayers. Phys. Chem. Chem. Phys. 18, 32560–32569. 10.1039/C6CP04883H 27874109

[B12] Célerse F. Lagardère L. Derat É. Piquemal J.-P. (2019). massively parallel implementation of steered molecular dynamics in tinker-hp: comparisons of polarizable and non-polarizable simulations of realistic systems. J. Chem. Theory Comput. 15, 3694–3709. 10.1021/acs.jctc.9b00199 31059250

[B13] Chowdhary J. Harder E. Lopes P. E. M. Huang L. MacKerell A. D. Roux B. (2013a). A polarizable force field of dipalmitoylphosphatidylcholine based on the classical drude model for molecular dynamics simulations of lipids. J. Phys. Chem. B 117, 9142–9160. 10.1021/jp402860e 23841725 PMC3799809

[B14] Chowdhary J. Li H. Harder E. Lopes P. MacKerell A. Roux B. (2013b). Drude model based polarizable force field for lipids. Biophys. J. 104:31a 10.1016/j.bpj.2012.11.210 PMC3799809 23841725

[B15] Cieplak P. Dupradeau F.-Y. Duan Y. Wang J. (2009). Polarization effects in molecular mechanical force fields. J. Phys. Condens. Matter 21:333102. 10.1088/0953-8984/21/33/333102 21828594 PMC4020598

[B16] Dang L. X. (1998). Importance of polarization effects in modeling the hydrogen bond in water using classical molecular dynamics techniques. J. Phys. Chem. B 102, 620–624. 10.1021/jp9731258

[B17] Das A. K. Urban L. Leven I. Loipersberger M. Aldossary A. Head-Gordon M.. (2019). Development of an advanced force field for water using variational energy decomposition analysis. J. Chem. Theory Comput. 15, 5001–5013. 10.1021/acs.jctc.9b00478 31408601

[B18] Debiec K. T. Gronenborn A. M. Chong L. T. (2014). Evaluating the strength of salt bridges: a comparison of current biomolecular force fields. J. Phys. Chem. B 118, 6561–6569. 10.1021/jp500958r 24702709 PMC4064690

[B19] Dill K. A. Bromberg S. Yue K. Fiebig K. M. Yee D. P. Thomas P. D.. (1995). Principles of protein folding–a perspective from simple exact models. Protein Sci. 4, 561–602. 10.1002/pro.5560040401 7613459 PMC2143098

[B20] Duboué-Dijon E. Delcroix P. Martinez-Seara H. Hladílková J. Coufal P. Křížek T.. (2018a). Binding of divalent cations to insulin: capillary electrophoresis and molecular simulations. J. Phys. Chem. B 122, 5640–5648. 10.1021/acs.jpcb.7b12097 29360367

[B21] Duboué-Dijon E. Mason P. E. Fischer H. E. Jungwirth P. (2018b). Hydration and ion pairing in aqueous Mg2 and Zn2 solutions: force-field description aided by neutron scattering experiments and ab initio molecular dynamics simulations. J. Phys. Chem. B 122, 3296–3306. 10.1021/acs.jpcb.7b09612 29116789

[B22] Duke R. E. Starovoytov O. N. Piquemal J.-P. Cisneros G. A. (2014). GEM^*^: a molecular electronic density-based force field for molecular dynamics simulations. J. Chem. Theory Comput. 10, 1361–1365. 10.1021/ct500050p 26580355 PMC5207213

[B23] Egberts E. Marrink S. J. Berendsen H. J. C. (1994). Molecular dynamics simulation of a phospholipid membrane. Eur. Biophys. J. 22, 423–436. 10.1007/BF00180163 8149924

[B24] Eisenberg M. Gresalfi T. Riccio T. McLaughlin S. (1979). Adsorption of monovalent cations to bilayer membranes containing negative phospholipids. Biochemistry 18, 5213–5223. 10.1021/bi00590a028 115493

[B25] Feigenson G. W. (1986). On the nature of calcium ion binding between phosphatidylserine lamellae. Biochemistry 25, 5819–5825. 10.1021/bi00367a071 3778883

[B26] Fitch C. A. Karp D. A. Lee K. K. Stites W. E. Lattman E. E. Bertrand García-Moreno E. (2002). Experimental pKa values of buried residues: analysis with continuum methods and role of water penetration. Biophys. J. 82, 3289–3304. 10.1016/S0006-3495(02)75670-1 12023252 PMC1302117

[B27] Freddolino P. L. Harrison C. B. Liu Y. Schulten K. (2010). Challenges in protein folding simulations: timescale, representation, and analysis. Nat. Phys. 6, 751–758. 10.1038/nphys1713 21297873 PMC3032381

[B28] Freddolino P. L. Park S. Roux B. Schulten K. (2009). Force field bias in protein folding simulations. Biophys. J. 96, 3772–3780. 10.1016/j.bpj.2009.02.033 19413983 PMC2711430

[B29] Friesner R. A. (2005). Modeling polarization in proteins and protein–ligand complexes: methods and preliminary results, in Advances in Protein Chemistry. eds Baldwin R. L. Baker D. (Amsterdam: Academic Press), 79–104. 10.1016/S0065-3233(05)72003-9 16581373

[B30] García-Moreno B. Dwyer J. J. Gittis A. G. Lattman E. E. Spencer D. S. Stites W. E. (1997). Experimental measurement of the effective dielectric in the hydrophobic core of a protein. Biophys. Chem. 64, 211–224. 10.1016/S0301-4622(96)02238-7 9127946

[B31] Gresh N. Cisneros G. A. Darden T. A. Piquemal J.-P. (2007). Anisotropic, polarizable molecular mechanics studies of inter- and intramolecular interactions and ligand-macromolecule complexes. A Bottom-Up Strategy. J. Chem. Theory Comput. 3, 1960–1986. 10.1021/ct700134r 18978934 PMC2367138

[B32] Gresh N. Perahia D. de Courcy B. Foret J. Roux C. El-Khoury L.. (2016). Complexes of a Zn-metalloenzyme binding site with hydroxamate-containing ligands. A case for detailed benchmarkings of polarizable molecular mechanics/dynamics potentials when the experimental binding structure is unknown. J. Comput. Chem. 37, 2770–2782. 10.1002/jcc.24503 27699809

[B33] Gresh N. Piquemal J.-P. Krauss M. (2005). Representation of Zn(II) complexes in polarizable molecular mechanics. Further refinements of the electrostatic and short-range contributions. Comparisons with parallel *ab initio* computations. J. Comput. Chem. 26, 1113–1130. 10.1002/jcc.20244 15934064

[B34] Harayama T. Riezman H. (2018). Understanding the diversity of membrane lipid composition. Nat. Rev. Mol. Cell Biol. 19, 281–296. 10.1038/nrm.2017.138 29410529

[B35] Harder E. Mackerell A. D. Jr. Roux B. (2009). Many-body polarization effects and the membrane dipole potential. J. Am. Chem. Soc. 131, 2760–2761. 10.1021/ja806825g 19199514 PMC2880651

[B36] Harger M. Li D. Wang Z. Dalby K. Lagardère L. Piquemal J.-P.. (2017). Tinker-OpenMM: absolute and relative alchemical free energies using AMOEBA on GPUs. J. Comput. Chem. 38, 2047–2055. 10.1002/jcc.24853 28600826 PMC5539969

[B37] Hauser H. Hinckley C. C. Krebs J. Levine B. A. Phillips M. C. Williams R. J. (1977). The interaction of ions with phosphatidylcholine. Biochim. Biophys. Acta 468, 364–377. 10.1016/0005-2736(77)90288-7 884090

[B38] Huang J. Lemkul J. A. Eastman P. K. MacKerell A. D. Jr. (2018). Molecular dynamics simulations using the drude polarizable force field on GPUs with OpenMM: Implementation, validation, and benchmarks. J. Comput. Chem. 39, 1682–1689. 10.1002/jcc.25339 29727037 PMC6031474

[B39] Huang J. MacKerell A. D. Jr. (2013). CHARMM36 all-atom additive protein force field: validation based on comparison to NMR data. J. Comput. Chem. 34, 2135–2145. 10.1002/jcc.23354 23832629 PMC3800559

[B40] Huang J. MacKerell A. D. Jr. (2014). Induction of peptide bond dipoles drives cooperative helix formation in the (AAQAA)3 peptide. Biophys. J. 107, 991–997. 10.1016/j.bpj.2014.06.038 25140435 PMC4142245

[B41] Huang J. Simmonett A. C. Pickard F. C. IV. MacKerell A. D. Jr. Brooks B. R. (2017). Mapping the drude polarizable force field onto a multipole and induced dipole model. J. Chem. Phys. 147:161702. 10.1063/1.4984113 29096511 PMC5459616

[B42] Javanainen M. Melcrová A. A. Magarkar A. Jurkiewicz P. Hof M. Jungwirth P.. (2017). Two cations, two mechanisms: interactions of sodium and calcium with zwitterionic lipid membranes. Chem. Commun. 53, 5380–5383. 10.1039/C7CC02208E 28453006

[B43] Jiang W. Hardy D. J. Phillips J. C. Mackerell A. D. Jr. Schulten K. Roux B. (2011). High-performance scalable molecular dynamics simulations of a polarizable force field based on classical Drude oscillators in NAMD. J. Phys. Chem. Lett. 2, 87–92. 10.1021/jz101461d 21572567 PMC3092300

[B44] Jiao D. Golubkov P. A. Darden T. A. Ren P. (2008). Calculation of protein–ligand binding free energy by using a polarizable potential. Proc. Natl. Acad. Sci. U.S.A. 105, 6290–6295. 10.1073/pnas.0711686105 18427113 PMC2359813

[B45] Jing Z. Liu C. Cheng S. Y. Qi R. Walker B. D. Piquemal J.-P.. (2019). Polarizable force fields for biomolecular simulations: recent advances and applications. Annu. Rev. Biophys. 48, 371–394. 10.1146/annurev-biophys-070317-033349 30916997 PMC6520134

[B46] Jing Z. Liu C. Qi R. Ren P. (2018). Many-body effect determines the selectivity for Ca and Mg in proteins. Proc. Natl. Acad. Sci. U.S.A. 115, E7495–E7501. 10.1073/pnas.1805049115 30038003 PMC6094099

[B47] Jolly L. H. Duran A. Lagardère L. Ponder J. W. Ren P. Y. Piquemal J.-P. (2019). Raising the performance of the Tinker-HP molecular modeling package [article v1.0]. LiveCoMS 1:10409 10.33011/livecoms.1.2.10409

[B48] Jorgensen W. L. (2007). Special issue on polarization. J. Chem. Theory Comput. 3:1877. 10.1021/ct700252g 26636192

[B49] Kleshchonok A. Tkatchenko A. (2018). Tailoring van der Waals dispersion interactions with external electric charges. Nat. Commun. 9:3017. 10.1038/s41467-018-05407-x 30069005 PMC6070553

[B50] Kohagen M. Mason P. E. Jungwirth P. (2014). Accurate description of calcium solvation in concentrated aqueous solutions. J. Phys. Chem. B 118, 1–27. 10.1021/jp5005693 24802184

[B51] Kohagen M. Pluharová E. Mason P. E. Jungwirth P. (2015). Exploring ion-ion interactions in aqueous solutions by a combination of molecular dynamics and neutron scattering. J. Phys. Chem. Lett. 6, 1563–1567. 10.1021/acs.jpclett.5b00060 26263314

[B52] Kurland R. Newton C. Nir S. Papahadjopoulos D. (1979). Specificity of Na+ binding to phosphatidylserine vesicles from a 23Na NMR relaxation rate study. Biochim. Biophys. Acta 551, 137–147. 10.1016/0005-2736(79)90360-2 427149

[B53] Lagardère L. Aviat F. Piquemal J.-P. (2019). Pushing the limits of multiple-time-step strategies for polarizable point dipole molecular dynamics. J. Phys. Chem. Lett. 10, 2593–2599. 10.1021/acs.jpclett.9b00901 31050904

[B54] Lagardère L. Jolly L.-H. Lipparini F. Aviat F. Stamm B. Jing Z. F.. (2018). Tinker-HP: a massively parallel molecular dynamics package for multiscale simulations of large complex systems with advanced point dipole polarizable force fields. Chem. Sci. 9, 956–972. 10.1039/C7SC04531J 29732110 PMC5909332

[B55] Lagardère L. Lipparini F. Polack É. Stamm B. Cancès É. Schnieders M.. (2015). Scalable evaluation of polarization energy and associated forces in polarizable molecular dynamics: II. Toward massively parallel computations using smooth particle mesh Ewald. J. Chem. Theory Comput. 11, 2589–2599. 10.1021/acs.jctc.5b00171 26575557

[B56] Lamoureux G. MacKerell A. D. Jr. Roux B. (2003). A simple polarizable model of water based on classical Drude oscillators. J. Chem. Phys. 119, 5185–5197. 10.1063/1.1598191 PMC3562330 23343286

[B57] Lee A. G. (2004). How lipids affect the activities of integral membrane proteins. Biochim. Biophys. Acta 1666, 62–87. 10.1016/j.bbamem.2004.05.012 15519309

[B58] Lee S. Park S. S. (2011). Dielectric properties of organic solvents from non-polarizable molecular dynamics simulation with electronic continuum model and density functional theory. J. Phys. Chem. B 115, 12571–12576. 10.1021/jp207658m 21967704

[B59] Lemkul J. A. Huang J. Roux B. Mackerell A. D. (2016). An empirical polarizable force field based on the classical drude oscillator model: development history and recent applications. Chem. Rev. 116, 4983–5013. 10.1021/acs.chemrev.5b00505 26815602 PMC4865892

[B60] Leontyev I. Stuchebrukhov A. (2011). Accounting for electronic polarization in non-polarizable force fields. Phys. Chem. Chem. Phys. 13:2613. 10.1039/c0cp01971b 21212894

[B61] Leontyev I. V. Stuchebrukhov A. A. (2009). Electronic continuum model for molecular dynamics simulations. J. Chem. Phys. 130:085102. 10.1063/1.3060164 19256627 PMC3910273

[B62] Leontyev I. V. Stuchebrukhov A. A. (2010a). Electronic continuum model for molecular dynamics simulations of biological molecules. J. Chem. Theory Comput. 6, 1498–1508. 10.1021/ct9005807 25364313 PMC4213183

[B63] Leontyev I. V. Stuchebrukhov A. A. (2010b). Electronic polarizability and the effective pair potentials of water. J. Chem. Theory Comput. 6, 3153–3161. 10.1021/ct1002048 25383062 PMC4224308

[B64] Leontyev I. V. Stuchebrukhov A. A. (2012). Polarizable mean-field model of water for biological simulations with amber and charmm force fields. J. Chem. Theory Comput. 8, 3207–3216. 10.1021/ct300011h 25580096 PMC4285689

[B65] Lipparini F. Lagardère L. Stamm B. Cancès E. Schnieders M. Ren P. (2014). Scalable Evaluation of Polarization Energy and Associated Forces in Polarizable Molecular Dynamics: I. Toward Massively Parallel Direct Space Computations. J. Chem. Theory Comput. 10, 1638–1651. 10.1021/ct401096t 26512230 PMC4620587

[B66] Liu C. Piquemal J.-P. Ren P. (2019). AMOEBA+ classical potential for modeling molecular interactions. J. Chem. Theory Comput. 15, 4122–4139. 10.1021/acs.jctc.9b00261 31136175 PMC6615954

[B67] Loco D. Lagardère L. Caprasecca S. Lipparini F. Mennucci B. Piquemal J.-P. (2017). Hybrid QM/MM molecular dynamics with AMOEBA polarizable embedding. J. Chem. Theory Comput. 13, 4025–4033. 10.1021/acs.jctc.7b00572 28759205

[B68] Loco D. Lagardère L. Cisneros G. A. Scalmani G. Frisch M. Lipparini F.. (2019). Towards large scale hybrid QM/MM dynamics of complex systems with advanced point dipole polarizable embeddings. Chem. Sci. 10, 7200–7211. 10.1039/C9SC01745C 31588288 PMC6677116

[B69] Loco D. Polack É. Caprasecca S. Lagardère L. Lipparini F. Piquemal J.-P.. (2016). A QM/MM approach using the AMOEBA polarizable embedding: from ground state energies to electronic excitations. J. Chem. Theory Comput. 12, 3654–3661. 10.1021/acs.jctc.6b00385 27340904

[B70] Lopes P. E. M. Guvench O. MacKerell A. D. Jr. (2015). Current status of protein force fields for molecular dynamics simulations. Methods Mol. Biol. 1215, 47–71. 10.1007/978-1-4939-1465-4_3 25330958 PMC4554537

[B71] Lopes P. E. M. Huang J. Shim J. Luo Y. Li H. Roux B. (2013). Polarizable force field for peptides and proteins based on the classical drude oscillator. J. Chem. Theory Comput. 9, 5430–5449. 10.1021/ct400781b 24459460 PMC3896220

[B72] Lopes P. E. M. Roux B. Mackerell A. D. Jr. (2009). Molecular modeling and dynamics studies with explicit inclusion of electronic polarizability. Theory and applications. Theor. Chem. Acc. 124, 11–28. 10.1007/s00214-009-0617-x 20577578 PMC2888514

[B73] Lucas T. R. Bauer B. A. Patel S. (2012). Charge equilibration force fields for molecular dynamics simulations of lipids, bilayers, and integral membrane protein systems. BBA - Biomembranes 1818, 318–329. 10.1016/j.bbamem.2011.09.016 21967961 PMC4216680

[B74] Lund M. Vacha R. Jungwirth P. (2008). Specific ion binding to macromolecules: effects of hydrophobicity and ion pairing. Langmuir 24, 3387–3391. 10.1021/la7034104 18294017

[B75] Magarkar A. Jurkiewicz P. Allolio C. Hof M. Jungwirth P. (2017). Increased binding of calcium ions at positively curved phospholipid membranes. J. Phys. Chem. Lett. 8, 518–523. 10.1021/acs.jpclett.6b02818 28067523

[B76] Maier J. A. Martinez C. Kasavajhala K. Wickstrom L. Hauser K. E. Simmerling C. (2015). ff14SB: improving the accuracy of protein side chain and backbone parameters from ff99SB. J. Chem. Theory Comput. 11, 3696–3713. 10.1021/acs.jctc.5b00255 26574453 PMC4821407

[B77] Martinek T. Duboué-Dijon E. Timr Š. Mason P. E. Baxová K. Fischer H. E.. (2018). Calcium ions in aqueous solutions: Accurate force field description aided by *ab initio* molecular dynamics and neutron scattering. J. Chem. Phys. 148:222813. 10.1063/1.5006779 29907056

[B78] Mason P. E. Jungwirth P. Duboué-Dijon E. (2019). Quantifying the strength of a salt bridge by neutron scattering and molecular dynamics. J. Phys. Chem. Lett. 10, 3254–3259. 10.1021/acs.jpclett.9b01309 31125523

[B79] Mason P. E. Wernersson E. Jungwirth P. (2012). Accurate description of aqueous carbonate ions: an effective polarization model verified by neutron scattering. J. Phys. Chem. B 116, 8145–8153. 10.1021/jp3008267 22630587

[B80] Mattai J. Hauser H. Demel R. A. Shipley G. G. (1989). Interactions of metal ions with phosphatidylserine bilayer membranes: effect of hydrocarbon chain unsaturation. Biochemistry 28, 2322–2330. 10.1021/bi00431a051 2541783

[B81] Melcr J. Ferreira T. Jungwirth P. Ollila O. H. S. (2019). Improved Cation Binding to Lipid Bilayer With Negatively Charged POPS by Effective Inclusion of Electronic Polarization. Preprint manuscript at Zenodo.org. 31762275 10.1021/acs.jctc.9b00824

[B82] Melcr J. Martinez-Seara H. Nencini R. Kolafa J. Jungwirth P. Ollila O. H. S. (2018). Accurate binding of sodium and calcium to a POPC bilayer by effective inclusion of electronic polarization. J. Phys. Chem. B 122, 4546–4557. 10.1021/acs.jpcb.7b12510 29608850

[B83] Melcrová A. Pokorná S. Pullanchery S. Kohagen M. Jurkiewicz P. Hof M.. (2016). The complex nature of calcium cation interactions with phospholipid bilayers. Sci. Rep. 6:38035. 10.1038/srep38035 27905555 PMC5131315

[B84] Morozov A. V. Tsemekhman K. Baker D. (2006). Electron density redistribution accounts for half the cooperativity of alpha helix formation. J. Phys. Chem. B 110, 4503–4505. 10.1021/jp057161f 16526672

[B85] Mu X. Wang Q. Wang L.-P. Fried S. D. Piquemal J.-P. Dalby K. N.. (2014). Modeling organochlorine compounds and the σ-hole effect using a polarizable multipole force field. J. Phys. Chem. B 118, 6456–6465. 10.1021/jp411671a 24484473 PMC4065202

[B86] Narth C. Lagardère L. Polack É. Gresh N. Wang Q. Bell D. R.. (2016). Scalable improvement of SPME multipolar electrostatics in anisotropic polarizable molecular mechanics using a general short-range penetration correction up to quadrupoles. J. Comput. Chem. 37, 494–506. 10.1002/jcc.24257 26814845 PMC4730919

[B87] Patel S. Davis J. E. Bauer B. A. (2009). Exploring ion permeation energetics in gramicidin A using polarizable charge equilibration force fields. J. Am. Chem. Soc. 131, 13890–13891. 10.1021/ja902903m 19788320 PMC2818143

[B88] Pegado L. Marsalek O. Jungwirth P. Wernersson E. (2012). Solvation and ion-pairing properties of the aqueous sulfate anion: explicit versus effective electronic polarization. Phys. Chem. Chem. Phys. 14, 10248–10257. 10.1039/c2cp40711f 22728656

[B89] Phillips J. C. Zheng G. Kumar S. Kale L. V. (2002). NAMD: biomolecular simulation on thousands of processors. In: *ACM/IEEE SC 2002 Conference (SC'02)* (Baltimore, MD). 10.1109/SC.2002.10019

[B90] Piana S. Klepeis J. L. Shaw D. E. (2014). Assessing the accuracy of physical models used in protein-folding simulations: quantitative evidence from long molecular dynamics simulations. Curr. Opin. Struct. Biol. 24, 98–105. 10.1016/j.sbi.2013.12.006 24463371

[B91] Piana S. Lindorff-Larsen K. Shaw D. E. (2011). How robust are protein folding simulations with respect to force field parameterization? Biophys. J. 100, L47–L49. 10.1016/j.bpj.2011.03.051 21539772 PMC3149239

[B92] Piquemal J.-P. Chevreau H. Gresh N. (2007). Toward a separate reproduction of the contributions to the hartree-fock and DFT intermolecular interaction energies by polarizable molecular mechanics with the SIBFA potential. J. Chem. Theory Comput. 3, 824–837. 10.1021/ct7000182 26627402

[B93] Piquemal J.-P. Cisneros G. (2016). Status of the gaussian electrostatic model, a density-based polarizable force field, in Many-Body Effects and Electrostatics in Biomolecules, eds Cui Q. Ren P. Meuwly M. (Pan Stanford Publishing), 269–299. 10.1201/b21343-11

[B94] Piquemal J.-P. Cisneros G. A. Reinhardt P. Gresh N. Darden T. A. (2006a). Towards a force field based on density fitting. J. Chem. Phys. 124:104101. 10.1063/1.2173256 16542062 PMC2080832

[B95] Piquemal J.-P. Gresh N. Giessner-Prettre C. (2003). Improved formulas for the calculation of the electrostatic contribution to the intermolecular interaction energy from multipolar expansion of the electronic distribution. J. Phys. Chem. A 107, 10353–10359. 10.1021/jp035748t 26313624

[B96] Piquemal J.-P. Jordan K. D. (2017). Preface: special topic: from quantum mechanics to force fields. J. Chem. Phys. 147:161401. 10.1063/1.5008887 29096449

[B97] Piquemal J.-P. Perera L. Cisneros G. A. Ren P. Pedersen L. G. Darden T. A. (2006b). Towards accurate solvation dynamics of divalent cations in water using the polarizable amoeba force field: From energetics to structure. J. Chem. Phys. 125:054511. 10.1063/1.2234774 16942230

[B98] Pluhackova K. Kirsch S. A. Han J. Sun L. Jiang Z. Unruh T.. (2016). A critical comparison of biomembrane force fields: structure and dynamics of model DMPC, POPC, and POPE bilayers. J. Phys. Chem. B 120, 3888–3903. 10.1021/acs.jpcb.6b01870 27035634

[B99] Pluhařová E. Mason P. E. Jungwirth P. (2013). Ion pairing in aqueous lithium salt solutions with monovalent and divalent counter-anions. J. Phys. Chem. A 117, 11766–11773. 10.1021/jp402532e 23581250

[B100] Ponder J. W. Case D. A. (2003). Force fields for protein simulations. Adv. Protein Chem. 66, 27–85. 10.1016/S0065-3233(03)66002-X 14631816

[B101] Qi R. Jing Z. Liu C. Piquemal J.-P. Dalby K. N. Ren P. (2018). Elucidating the phosphate binding mode of phosphate-binding protein: the critical effect of buffer solution. J. Phys. Chem. B 122, 6371–6376. 10.1021/acs.jpcb.8b03194 29807433 PMC6173968

[B102] Rackers J. A. Ponder J. W. (2019). Classical Pauli repulsion: An anisotropic, atomic multipole model. J. Chem. Phys. 150:084104. 10.1063/1.5081060 30823770 PMC6386640

[B103] Rackers J. A. Wang Z. Lu C. Laury M. L. Lagardère L. Schnieders M. J.. (2018). Tinker 8: software tools for molecular design. J. Chem. Theory Comput. 14, 5273–5289. 10.1021/acs.jctc.8b00529 30176213 PMC6335969

[B104] Ren P. Ponder J. W. (2003). Polarizable atomic multipole water model for molecular mechanics simulation. J. Phys. Chem. B 107, 5933–5947. 10.1021/jp027815+

[B105] Rick S. W. Stuart S. J. (2003). Potentials and algorithms for incorporating polarizability in computer simulations, in Reviews in Computational Chemistry, eds Lipkowitz K. B. Boyd D. B. (Hoboken, NJ: John Wiley & Sons) 89–146.

[B106] Riera M. Lambros E. Nguyen T. T. Götz A. W. Paesani F. (2019). Low-order many-body interactions determine the local structure of liquid water. Chem. Sci. 116:7463 10.1039/C9SC03291F PMC6927411 32133122

[B107] Robertson M. J. Tirado-Rives J. Jorgensen W. L. (2015). Improved peptide and protein torsional energetics with the OPLSAA force field. J. Chem. Theory Comput. 11, 3499–3509. 10.1021/acs.jctc.5b00356 26190950 PMC4504185

[B108] Roux M. Bloom M. (1990). Calcium, magnesium, lithium, sodium, and potassium distributions in the headgroup region of binary membranes of phosphatidylcholine and phosphatidylserine as seen by deuterium NMR. Biochemistry 29, 7077–7089. 10.1021/bi00482a019 2223761

[B109] Roux M. Bloom M. (1991). Calcium binding by phosphatidylserine headgroups. Deuterium NMR study. Biophys. J. 60, 38–44. 10.1016/S0006-3495(91)82028-8 1883944 PMC1260036

[B110] Schröder C. (2012). Comparing reduced partial charge models with polarizable simulations of ionic liquids. Phys. Chem. Chem. Phys. 14, 3089–3102. 10.1039/c2cp23329k 22287020 PMC7613810

[B111] Shi Y. Jiao D. Schnieders M. J. Ren P. (2009). Trypsin-ligand binding free energy calculation with AMOEBA. Conf. Proc. IEEE Eng. Med. Biol. Soc. 2009, 2328–2331. 10.1109/IEMBS.2009.5335108 19965178 PMC2819397

[B112] Shi Y. Ren P. Schnieders M. Piquemal J.-P. (2015). Polarizable force fields for biomolecular modeling: parrill/reviews in computational chemistry volume 28, in Reviews in Computational Chemistry Volume 28 Reviews in Computational Chemistry, eds Parrill A. L. Lipkowitz K. B. (Hoboken, NJ: John Wiley and Sons, Inc.), 51–86. 10.1002/9781118889886.ch2

[B113] Shi Y. Xia Z. Zhang J. Best R. Wu C. Ponder J. W.. (2013). The Polarizable atomic multipole-based AMOEBA force field for proteins. J. Chem. Theory Comput. 9, 4046–4063. 10.1021/ct4003702 24163642 PMC3806652

[B114] Stone A. (2013). The Theory of Intermolecular Forces. Oxford 10.1093/acprof:oso/9780199672394.001.0001

[B115] Vacha R. Siu S. W. I. Petrov M. Böckmann R. A. Barucha-Kraszewska J. J. Jurkiewicz P.. (2009). Effects of alkali cations and halide anions on the DOPC lipid membrane. J. Phys. Chem. A 113, 7235–7243. 10.1021/jp809974e 19290591

[B116] Van Der Spoel D. Lindahl E. Hess B. Groenhof G. Mark A. E. Berendsen H. J. C. (2005). GROMACS: fast, flexible, and free. J. Comput. Chem. 26, 1701–1718. 10.1002/jcc.20291 16211538

[B117] Vazdar M. Jungwirth P. Mason P. E. (2013). Aqueous guanidinium–carbonate interactions by molecular dynamics and neutron scattering: relevance to ion–protein interactions. J. Phys. Chem. B 117, 1844–1848. 10.1021/jp310719g 23245268

[B118] Venable R. M. Krämer A. Pastor R. W. (2019). Molecular dynamics simulations of membrane permeability. Chem. Rev. 119, 5954–5997. 10.1021/acs.chemrev.8b00486 30747524 PMC6506413

[B119] Wang Q. Rackers J. A. He C. Qi R. Narth C. Lagardère L.. (2015). General model for treating short-range electrostatic penetration in a molecular mechanics force field. J. Chem. Theory Comput. 11, 2609–2618. 10.1021/acs.jctc.5b00267 26413036 PMC4570253

[B120] Wang W. Skeel R. D. (2005). Fast evaluation of polarizable forces. J. Chem. Phys. 123:164107. 10.1063/1.2056544 16268681

[B121] Wu J. C. Piquemal J.-P. Chaudret R. Reinhardt P. Ren P. (2010). Polarizable molecular dynamics simulation of Zn(II) in water using the AMOEBA force field. J. Chem. Theory Comput. 6, 2059–2070. 10.1021/ct100091j 21116445 PMC2992432

[B122] Zhang C. Lu C. Jing Z. Wu C. Piquemal J.-P. Ponder J. W.. (2018). AMOEBA polarizable atomic multipole force field for nucleic acids. J. Chem. Theory Comput. 14, 2084–2108. 10.1021/acs.jctc.7b01169 29438622 PMC5893433

[B123] Zhang J. Yang W. Piquemal J.-P. Ren P. (2012). Modeling structural coordination and ligand binding in zinc proteins with a polarizable potential. J. Chem. Theory Comput. 8, 1314–1324. 10.1021/ct200812y 22754403 PMC3383645

